# Inhibiting miR-155 protects against myocardial ischemia/reperfusion injury via targeted regulation of HIF-1α in rats

**DOI:** 10.22038/ijbms.2019.34853.8270

**Published:** 2019-09

**Authors:** Jian-Guo Chen, Xiao-Ming Xu, Hui Ji, Bo Sun

**Affiliations:** 1 Department of Pediatric Surgery, Jingzhou Central Hospital, the Second Clinical Medical College, Yangtze University, Jingzhou 434020, Hubei, China; 2 Department of Orthopedic surgery, Jingzhou Central Hospital, the Second Clinical Medical College, Yangtze University, Jingzhou 434020, Hubei, China; 3 Department of Clinical Laboratory, Jining Medical College Affiliated Hospital, Jining Medical College, Jining 272000, Shandong, China

**Keywords:** Alpha subunit, Apoptosis, Hypoxia-inducible factor 1, Ischemia, Mir-155 microRNA, human, Reperfusion

## Abstract

**Objective(s):**

The aim of this study was to identify the role of miR-155 in the myocardial ischemia/reperfusion (I/R) injury through targeting hypoxia-inducible factor 1-alpha (HIF-1α).

**Materials and Methods:**

We constructed rat models with myocardial I/R injury and H9C2 cell models with hypoxia/reoxygenation (H/R) damage. Anti-miR-155 and HIF-1α short hairpin RNA (shRNA) were used to treat rats and H9C2 cells to measure infarct area (IA) by TTC staining, determine creatine kinase (CK) and lactate dehydrogenase (LDH) activities by automatic biochemical analyzer, cardiac troponin T (cTnT) and cardiac troponin I (cTnI) levels by ELISA, and detect apoptosis-related proteins by Western blotting. TUNEL staining and flowcytometry were employed to evaluate the apoptosis, JC-1 staining to detect mitochondrial membrane potential (MMP), and MTT assay to determine H9C2 cell viability.

**Results:**

After I/R and H/R, significant elevations were observed in IA, apoptosis, CK, LDH, cTnT, cTnI, and miR-155 levels with reduced HIF-1α. Besides, H/R-induced H9C2 cells presented decreases in MMP and Bcl-2/Bax, but increases in cytosolic/mitochondrial ratio of cytochrome C (Cyt-C) and expressions of cleaved caspase-3 and cleaved caspase-9. However, both rats and H9C2 cells showed an opposite tendency concerning the above after anti-miR-155 treatment. Nevertheless, HIF-1α shRNA effectively reversed protective effects of anti-miR-155 on alleviating I/R- and H/R- induced injury.

**Conclusion:**

Inhibiting miR-155 could reduce myocardial infarct size, suppress I/R-induced cardiomyocyte apoptosis, and maintain the MMP to alleviate I/R-induced injury via specific regulation of HIF-1α.

## Introduction

Acute myocardial infarction (AMI) is still a common cause of death and disability for patients with the rapidly rising modality year by year, making it a great public health threat (1). At the time of myocardial ischemia or damage, the immediate reperfusion treatments, such as thrombolytic therapy, percutaneous coronary intervention (PCI), or immediate angioplasty constitute the most effective and life-saving measures to open the occluded coronary artery in patients with AMI (2). However, the rapid reperfusion would also result in further cardiomyocyte damage and impaired heart function, causing the myocardial ischemia-reperfusion (I-R) injury (3). According to previous studies, the Ca^2+ ^overload and overproduction of reactive oxygen species (ROS) produced by mitochondria, as well as granulocytes accumulation after endothelial injury during the reperfusion period would cause inflammatory responses, which damage the cellular membrane structures (4-6). However, the specific mechanism of I/R injury has not been clearly elucidated, and the effective treatment strategy is still a big challenge clinically (7, 8). MicroRNAs (miRNAs), a series of highly-reserved small non-coding RNA molecules with about 22 nucle

otides, were mainly discovered in eukaryotes (9). Through regulating the transcription of the target genes, miRNAs would participate in a variety of biological processes, including cell differentiation, proliferation and apoptosis (10). Increasing evidences have shown that miRNAs are critical in the pathogenesis of myocardial injury in humans, including myocardial I/R injury (11-13). For example, miR-155, as a typical multi-functional miRNA, is widely expressed in hematopoietic cells, fibroblasts, epithelial cells, and nerve cells (14, 15). In recent years, several lines of studies have identified that miR-155 was involved in atherosclerosis, hypertension, and ventricular hypertrophy (16, 17), and thus, it was recognized as a new intervention treatment for cardiovascular diseases. For instance, Yang *et al. *found that miR-155 could down-regulate its target gene (AGTR1) to inhibit calcium signaling pathway, thereby improving cardiac hypertrophy (18). Li and the colleagues reported that miR-155 can alleviate chronic inflammation via negative feedback loop, performing its protective anti-inflammatory functions in atherosclerosis-associated foam cell formation by mediating miR-155- calcium-regulated heat stable protein 1 (CARHSP 1)- tumor necrosis factor alpha (TNF-α) pathway (19). In addition, miR-155 could also target Forkhead box protein O3 a (FoxO3a) to mediate FoxO3a/ apoptosis repressor with caspase recruiting domain (ARC)/caspase-1 pathway in renal I/R injury, as demonstrated by Wu *et al *(20). Therefore, we hypothesized that miR-155 may also play an essential role in myocardial I/R injury.Notably, hypoxia-inducible factor 1-alpha (HIF-1α) was found to be the target gene of miR-155 by referring to a biological prediction website. Moreover, there was evidence to support that the cardio-protection strategies from ischemia, like ischemic preconditioning and ischemic postconditioning were performed through up-regulation of HIF-1α (21). Hence, we investigated whether miR-155 can play a regulatory role in myocardial I/R injury by targeting HIF-1α in this study, which could contribute to the clinical treatment of I/R injury.

## Materials and Methods

***Ethics statement***

 This study had the approval from the Ethics Committee for Animal Experiments in our hospital and was strictly obedience to the *Guide for the Care and Use of Laboratory Animals *published by the National Institute of Health in the USA (22).

***Establishment of rat models with myocardial I/R injury***

Fifty Sprague-Dawley (SD) rats of specific-pathogen-free (SPF) grade (250-300 g) were obtained from Shanghai Laboratory Animal Center with 10 rats per each group. The rats were anesthetized by Pentobarbital sodium (30 mg/kg; Sigma) and the left anterior descending coronary artery (LAD) ligation was performed to induce myocardial I/R rat models. At first, rats were fixed on the operation table in supine position, while the tracheal cannula was connected to the DH150 animal ventilator (Harvard Apparatus, USA), and the rats were monitored by the electrocardiographic electrodes mounted on limbs for electrocardiogram (ECG) recording (Niho Kohden, Japan). At the position about 2-3 mm to the lower and middle edge of auricula sinistra, a 6-0 silk suture was used for 30 min for ligation of LAD. Next, the ligature was removed, followed by 3 hr of reperfusion and then the chest was closed. All rats were equally randomized to five groups: Sham group (without ligation of LAD), I/R group (model rats with myocardial I/R injury), I/R + anti-miR-155 group, I/R + anti-control group, and I/R + anti-miR-155 + shHIF-1α group. Rats in the latter three groups received injection of 1x10^7 ^plaque forming units (PFU) anti-miR-155, anti-control and shHIF-1α lentivirus into six separate sites of the left ventricular anterior wall via a 26-gauge needle about four days before I/R model construction. Anti-miR-155, anti-control and shHIF-1α lentivirus were purchased from Shanghai Genechem Co, Ltd.

***Cell culture and H/R treatment***

H9C2 cells were purchased from the Cell bank of the Chinese Academy of Sciences (Shanghai, China). For hypoxia/ reoxygenation (H/R) treatment, cells were collected at the logarithmic growth phase and cultured for 12 hr in serum-free DMEN/F12 medium and replaced in glucose-free culture medium for 1 hr of incubation in a hypoxic chamber at 37 ^°^C (with 5% CO_2 _and 95% N_2_) and then transferred into normal culture medium for 3 hr of reoxygenation under the condition of 37 ^°^C and 5% CO_2_. Then, the H9C2 cells were divided into the following five groups: Control group (cells without any treatment), H/R group (cells with H/R treatment), H/R+anti-miR-155 group (cells transfected with anti-miR-155 lentivirus before H/R treatment), H/R+anti-control group (cells transfected with anti-control lentivirus before H/R treatment), and H/R+anti-miR-155+shHIF-1α group (cells transfected with anti-miR-155 and shHIF-1α lentiviruses before H/R treatment). The lentivirus transduction agent (Thermo Scientific) was applied with anti-miR-155, anti-control or HIF-1α short hairpin RNA (shRNA) according to the instruction of the manufacturer as for lentivirus transduction. H/R treatment should be performed about 48 hr after the transfection with lentivirus.

***Dual-luciferase reporter gene assay***

*HIF-1α* target gene sequences were inserted into wild-type HIF-1α-3’ untranslated region (UTR-WT) plasmid and mutant-type HIF-1α-3’UTR-MUT plasmid to construct *HIF-1α* dual-luciferase reporter gene plasmids. Next, 293T cells, harvested at the logarithmic growth phase, were inoculated onto 96-well plates. When cell density reached about 70%, Lipofectamine 2000 was used for cell transfection. After co-transfection with HIF-1α-3’UTR-WT or HIF-1α-3’UTR-MUT plasmids and miR-155 mimic/mimic negative control (NC) (Shanghai Genechem Co., Ltd.), 293T cells were cultured for 6 hr in an incubator (Thermo, USA) and then transferred into new medium containing 10% fetal bovine serum for 48 hr of continued incubation. Dual-luciferase reporter gene assay was conducted by following the instructions provided by Promega and the expression levels of reporter genes were expressed as the ratio of firefly to Renilla luciferase activity (Fluc/Rluc).

**TTC staining**

After reperfusion, rats were sacrificed and their heart were taken out and cryopreserved for 10 min at -80 ^°^C in a refrigerator. Along the longitudinal direction of the heart, 5-6 tissue sections in 2 mm-thickness from cardiac base to apex were obtained. Then, the tissue sections were incubated in in 1% triphenyl tetrazolium chloride (TTC) solution at 37°C for 15 min in dark. The sections were then fixed in 10% formaldehyde solution for 1 hr and photographed under natural light. The software Image-Pro 6.0 was used to analyze all areas, including area at risk (AAR), infarct area (IA) and left ventricle (LV) area. The ratio of IA/LV and IA/AAR were calculated.

***Determination of CK and LDH activities, and levels of cTnT***

After reperfusion, the arterial blood was extracted and H9C2 cells were collected from each group. After centrifugation for 10 min at the rate of 3000 g/min, serum was extracted for the determination of creatine kinase (CK) and lactate dehydrogenase (LDH) activities with an automatic biochemical analyzer (AU-2700, Olympus, Japan). Troponin T (cTnT) and troponin I (cTnI) levels were detected using rat ELISA Kit by microplate reader (MULTISKAN MK3; Thermo, San Jose, CA, USA). Each experiment was performed independently three times.

**qRT-PCR**

Myocardial tissues were ground with normal saline and total RNA was extracted by using an extraction kit (Omega, USA). UV spectrometer (UV-1800, Japan) was used to determine the purity and concentration of RNA and agarose gel electrophoresis to detect the intactness of RNA. Primer sequences of miR-155 and HIF-1α were designed by using Primer 5.0 and synthesized by Sangon Biotech (Shanghai) Co, Ltd. The primers of miR-155 used in the study were as follows: miR-155: F: 5’-ACACTCCAGCTGGGAGCTGGTGTTG-3’, R: 5’-GTGCAGGGTCCGAGGT-3’; U6: R: 5’-GTGTGCGGTCCGA

GGT-3’, F: 5’-GAAGGTGAAGGTCGGAGTC-3’. The reverse transcription of RNA into cDNA was performed with a PrimescriptTM RT reagent Kit (Takara, Japan). The process of qRT-PCR was completed by using a SYBR® premix Ex Taq TM kit (Takara Biotechnology Co, Ltd., Dalian, China). The relative expression of miR-155 was calculated by using 2 ^- ΔΔCt^ method with U6 as the internal reference gene. Each experiment was repeated independently at least three times. 

***MTT assay***

H9C2 cells were plated in 96-well plates, followed by addition of 20 μl of MTT (3-(4,5-dimethylthiazol-2-yl)2,5-diphenyl tetrazolium bromide) solution (5 mg/ml, Sigma, USA) and incubation for 4 hr. Subsequently, the culture fluid was removed and 150 μl of dimethyl sulfoxide (DMSO) was added for oscillation (10 min). At last, an enzyme-linked immunosorbent assay (ELISA) reader was used to determine the absorbance (OD) value at the wavelength of 490 nm, and cell viability was calculated according to the following formula: cell viability = OD value of experiment group/OD value of control group × 100%. The experiment was repeated for three times.

***TUNEL staining***

Intraperitoneal injection of pentobarbital sodium was performed to euthanize the rats. First, the chest was opened, 10 ml phosphate-buffered saline (PBS) was injected by retrograde through the aorta, and 4% paraformaldehyde was applied for reperfusion. Next, the heart of rats was taken out, fixed in 4% paraformaldehyde for 24 hr, dehydrated routinely and embedded in paraffin, sliced into 10 continuous sections of 3 μm in thickness, and baked at 50 ^°^C for 1 hr. Then, immunostaining permeabilization buffer (0.1% Triton X-100 dissolved in 0.1% sodium citrate solution) was added for 2 min of incubation in ice bath. After washing with PBS buffer, sections were incubated with 50 μl of TUNEL (terminal deoxynucleotidyl transferase-mediated dUTP nick end-labeling) solution at 37 ^°^C in a wet box for 60 min. After that, 50 μl of DAPI (4’,6-diamidino-2-phenylindole) stains was added, and cells were incubated for 5 min at 37 °C in dark. The apoptotic cells were photographed and counted under a fluorescence microscope. Each experiment was repeated three times.

***Flow cytometry***

Single-cell suspension was re-suspended with a straw and placed in 1 × binding buffer, and cell density was adjusted to 1 × 10^6^ cells/ml. Next, 100 µl of cell suspension was added into a glass centrifuge tube, followed by the addition of 5 µl of Annexin V-FITC and 10 µl of propidium iodide (PI) to incubate for 15 min at 20-25 ^°^C in avoidance of light, with the addition of 400 µl 1 × binding buffer lastly. The excitation wavelength was 355 nm, and the emission wavelength was 488 nm. The FACS and Cell Quest software were used for data collecting and analysis, and the percentage of positive cells was recorded. Each experiment was repeated three times.

***Mitochondrial membrane potential using JC-1 staining***

The working solution of JC-1 dye and cell culture medium were mixed together by 1:1, which was added into H9C2 cells from each group before the incubation of cells for 20 min at 37 ^°^C with CO_2_. The excess dye was removed by washing with JC-1staining buffer and then rinsed three times with PBS. Subsequently, cells were observed and photographed under a confocal laser scanning microscope, while the OD value under green fluorescence (490 nm/530 nm) and red fluorescence (525 nm/590 nm) was determined, respectively. The results were expressed as red/green fluorescence ratio. Each experiment was repeated three times.

***Western blotting***

The mitochondria and cytoplasm of H9C2 cells from each group were separated according to the protocol provided on the Cell Mitochondria Isolation Kit (Beyotime). Then, the mitochondria and cytoplasmic proteins were extracted, followed by 15 min of centrifugation at the rate of 12000 rpm, and the supernatant was collected for sodium dodecyl sulfate polyacrylamide gel electrophoresis (SDS-PAGE). The proteins were electrophoretically transferred to nitrocellulose membrane, which was placed in 5% skimmed milk-PBS buffer for 1 hr of blocking. Next, primary antibodies were added for overnight incubation at 4 ^°^C, including HIF-1α, Bcl-2, Bax, cytochrome C (Cyt-C), cleaved caspase-3, and cleaved caspase-9 (1:1000, Abcam, USA). Then, the membrane was washed with PBS buffer for three times and incubated with horseradish peroxidase (HRP)-conjugated secondary antibodies for 1 hr of incubation at room temperature. Finally, enhanced chemiluminescence method was used for development. Relative expression levels of proteins were presented as the gray value ratio of target band to reference band, with GAPDH as the internal reference gene. Each experiment was repeated three times.

***Statistical methods***

The SPSS 21.0 software was used for statistical data analysis. Measurement data was presented by mean ± standard deviation (`x±s). Inter-group comparison analysis was performed by Student’s t-test, and difference among multiple groups was tested by one-way ANOVA. *P*<0.05 indicated the significance of differences. 

## Results

***Determination of IA of rats***

**Figure 1:**
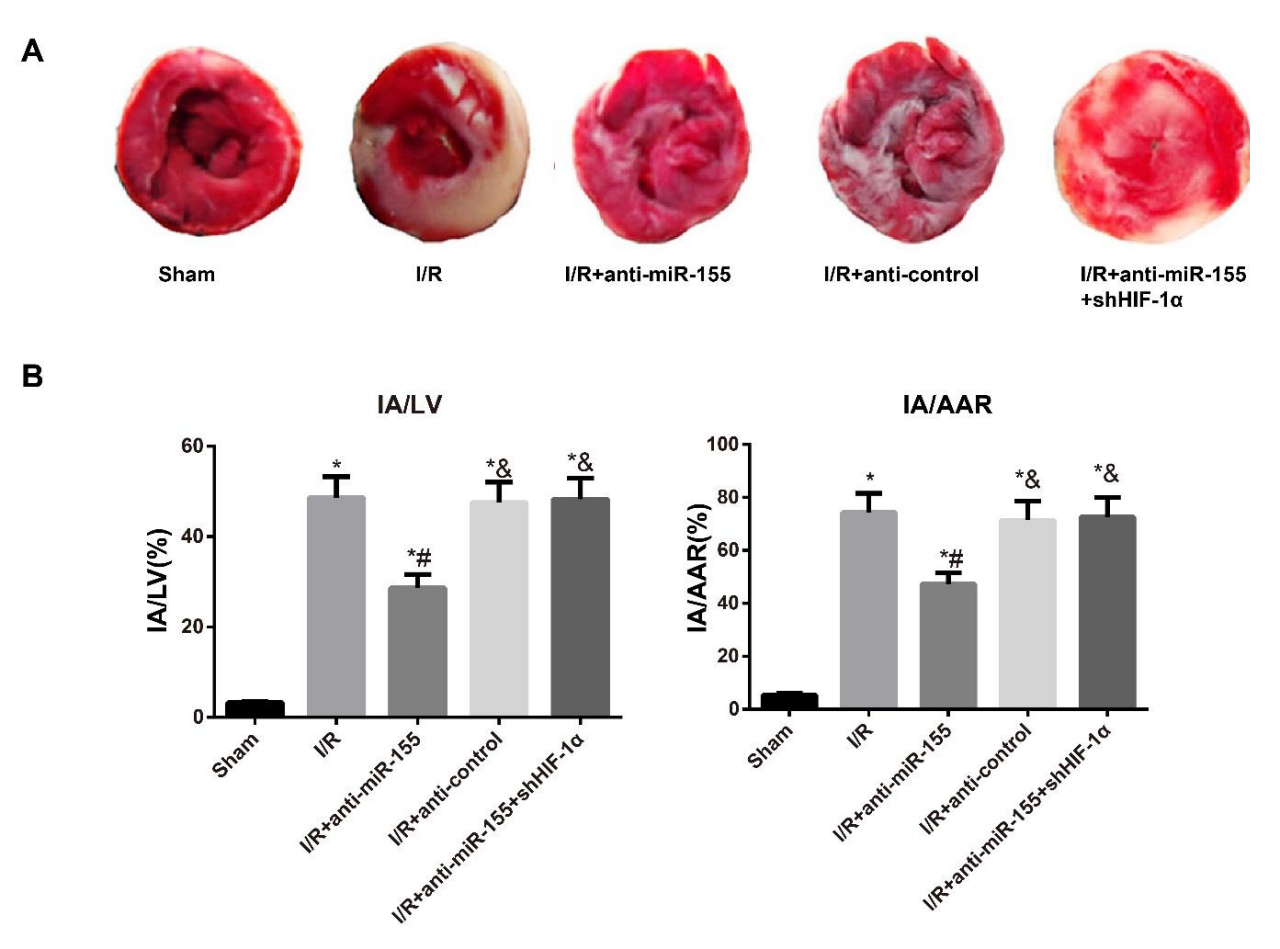
Determination of the infarct area of rats in each group

Note: A, Graph of IA of rats in each group; B, The IA/LV and IA/AAR ratios of rats in each group; *, *P*<0.05 compared to Sham group; #, *P*<0.05 compared to the I/R group; and, *P*<0.05 compared to the I/R+anti-miR-155 group. AAR: Area at risk, IA: Infarct area, LV: Left ventricle, I/R: Ischemia/reperfusion

As presented by Figure 1, the myocardial tissues in red were AAR, while those in white were IA. Compared to Sham tissues, those tissues in other groups had significantly increased ratios of IA/LV and IA/AAR (all *P*<0.05). Conversely, the ratios of IA/LV and IA/AAR were lower in tissues from the I/R+anti-miR-155 group than those from I/R group (all *P*<0.05), but no obvious differences were found between I/R and I/R+anti-control groups (all *P*>0.05). Additionally, the markedly elevated IA/LV and IA/AAR ratios were observed in tissues from the I/R+anti-miR-155+shHIF-1α group in comparison with the I/R+anti-miR-155 group (all *P*<0.05). 

**Figure 2:**
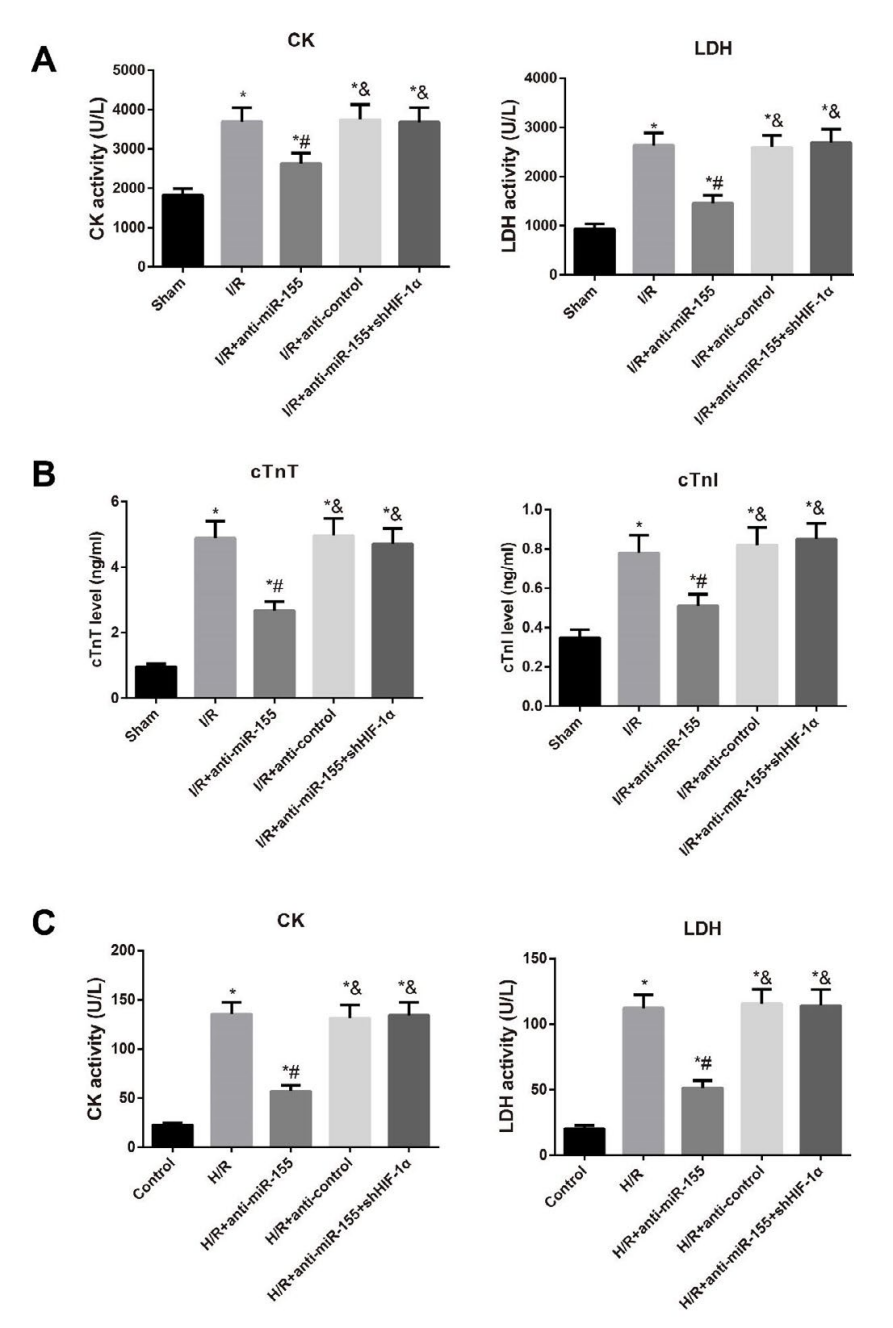
The levels of CK, LDH, cTnT and cTnI in the serum of rats (A, B) and H9C2 cells (C) of different groups

Note: *, *P*<0.05 compared to Sham/Control group; #, *P*<0.05 compared to the I/R or H/R group; and, *P*<0.05 compared to the I/R+anti-miR-155 or H/R+anti-miR-155 group. CK: Creatine kinase, LDH: Lactate dehydrogenase, cTnI: Cardiac troponin I, cTnT: Cardiac troponin T, I/R: Ischemia/reperfusion, H/R: Hypoxia/ reoxygenation

**Figure 3:**
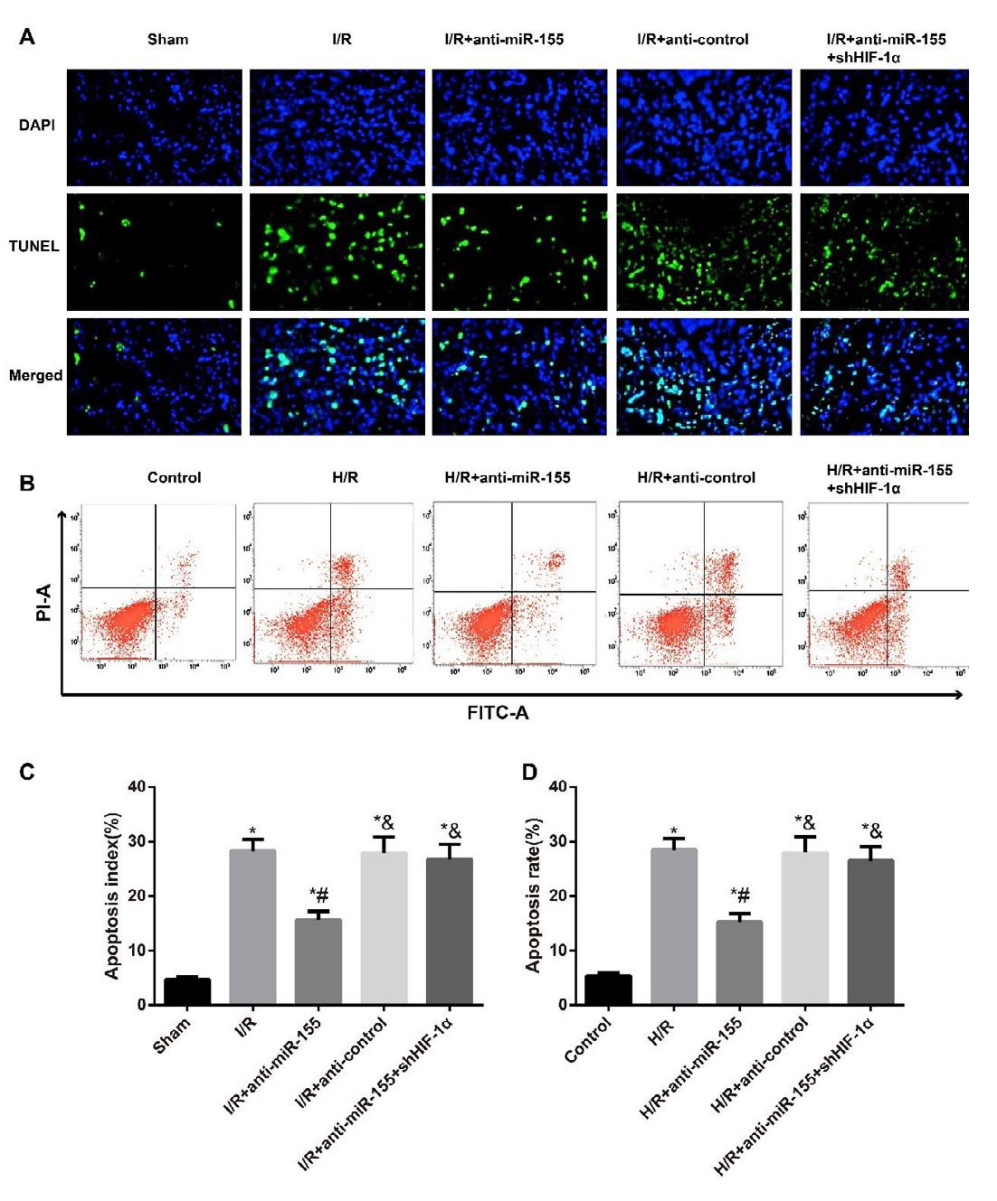
Apoptosis detection in the myocardial tissues of rats and H9C2 cells from different groups

Note: A, The apoptosis in the myocardial tissues of rats detected by TUNEL (terminal deoxynucleotidyl transferase-mediated dUTP nick end-labeling) assay; B, H9C2 cell apoptosis detected by flowcytometry; C-D, Comparison of the apoptosis rate in the myocardial tissues of rats (C) and H9C2 cells (D), respectively; *, *P*<0.05 compared to Sham/Control group; #, *P*<0.05 compared to the ischemia/reperfusion (I/R) or hypoxia/ reoxygenation (H/R) group; and, *P*<0.05 compared to the I/R+anti-miR-155 or H/R+anti-miR-155 group

**Figure 4:**
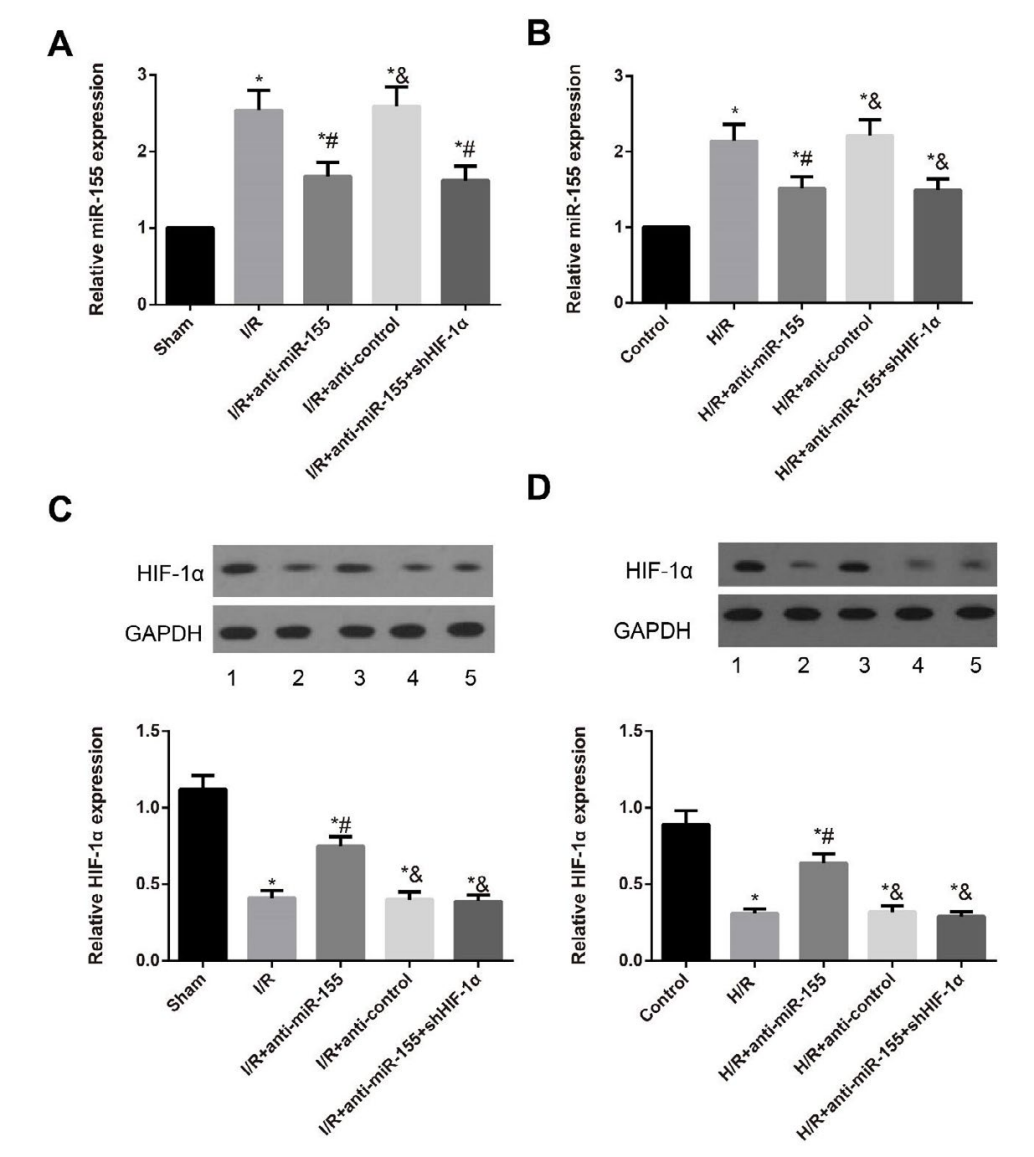
The expression of miR-155 and HIF-1α in rats and H9C2 cells from different groups

Note: A-B, The expression level of miR-155 in the myocardial tissues of rats (A) and H9C2 cells (B) detected by qRT-PCR; C-D, The protein expression of HIF-1α in the myocardial tissues of rats (C) and H9C2 cells (D) detected by Western blotting; *, *P*<0.05 compared to Sham/Control group; #, *P*< 0.05 compared to the I/R or H/R group; and, *P*<0.05 compared to the I/R+anti-miR-155 or H/R+anti-miR-155 group. HIF-1α: Hypoxia-inducible factor 1-alpha, I/R: Ischemia/reperfusion, H/R: Hypoxia/ reoxygenation

**Figure 5:**
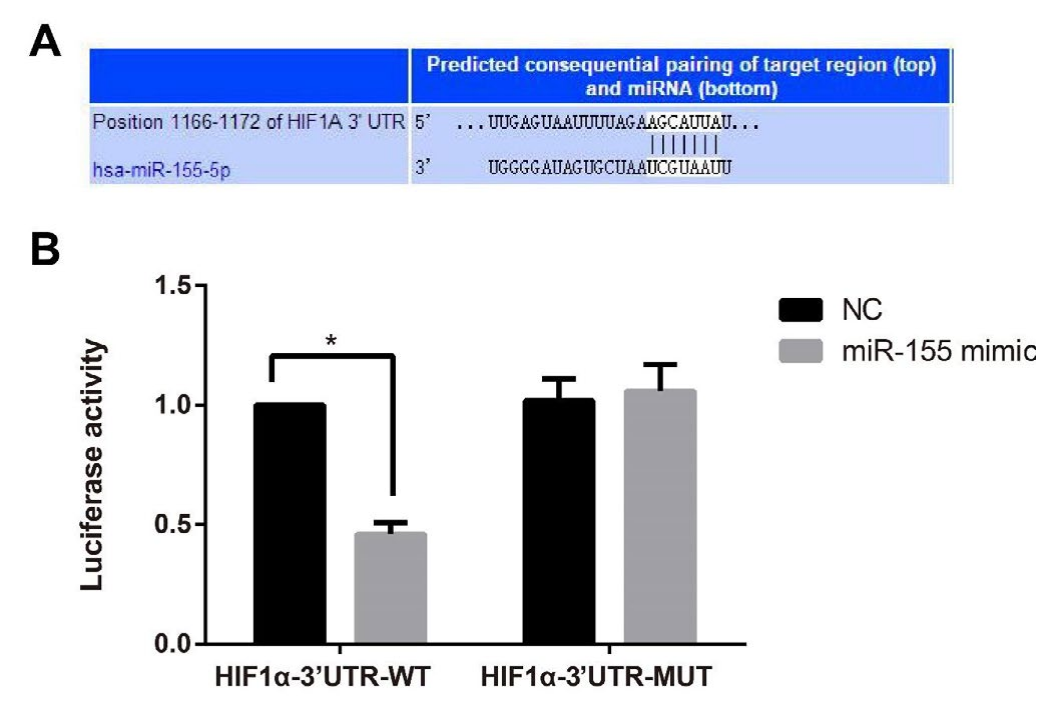
Verification of the targeting relationship between miR-155 and HIF-1α

Note: A, The binding site between miR-155 and the 3’-UTR (untranslated region) of hypoxia-inducible factor 1-alpha (HIF-1α) mRNA; B, Dual-luciferase reporter gene assay confirmed the targeting relationship between miR-155 and HIF-1α. *, *P*<0.05 suggested the significance of differences

**Figure 6:**
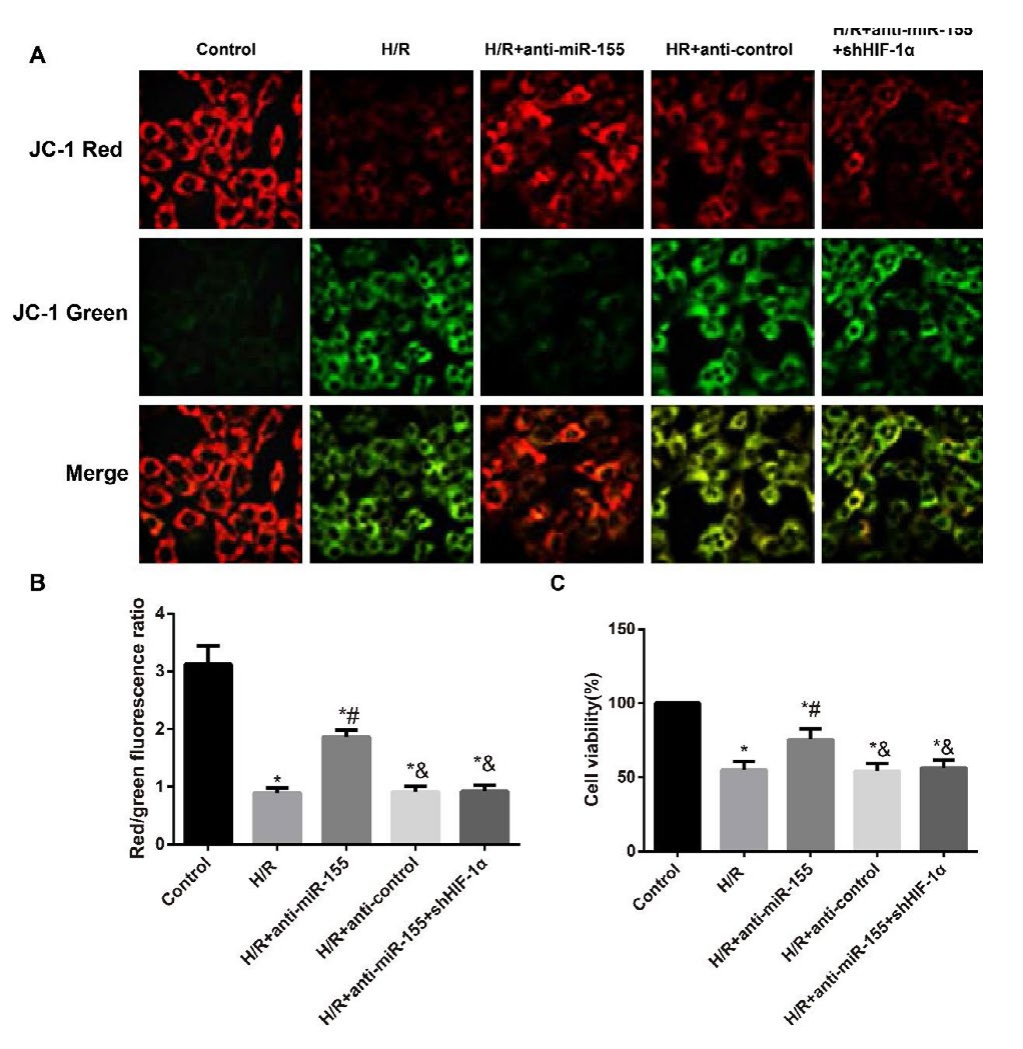
The determination of MMP and cell viability in H9C2 cells

Note: A, The determination of mitochondrial membrane potential (MMP) in H9C2 cells evaluated by JC-1 staining; B, The ratio of red/green fluorescence in H9C2 cells in each group; C, The viability of H9C2 cells in each group detected by MTT; *, *P*<0.05 compared to Control group; #, *P*<0.05 compared to the hypoxia/ reoxygenation (H/R) group; and, *P*<0.05 compared to the H/R+anti-miR-155 group

**Figure 7:**
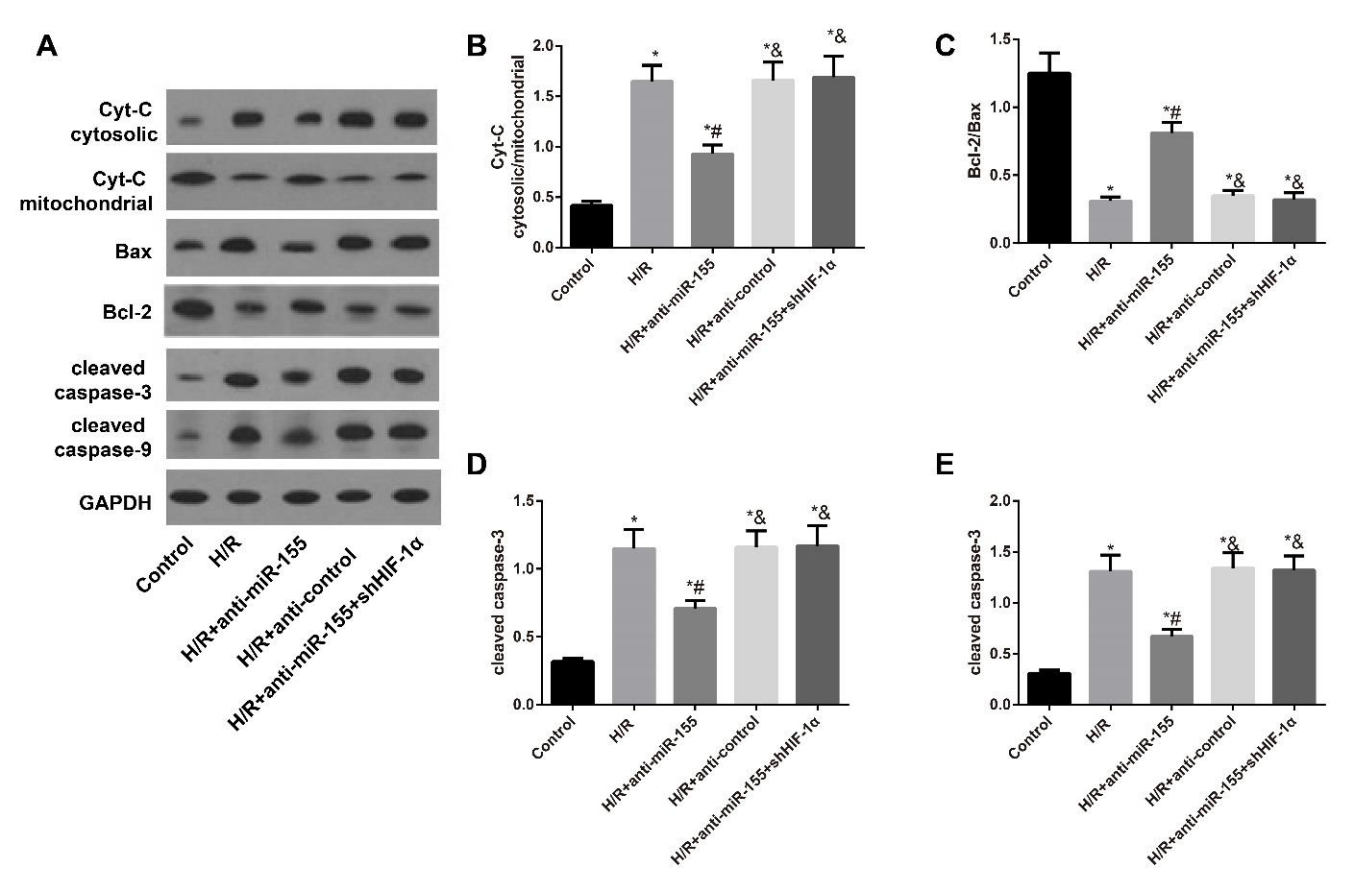
Expression of apoptosis-related proteins in H9C2 cells in each group

Note: A, The expression of apoptosis-related proteins in H9C2 cells in each group determined by Western blotting; B-E, The expression levels of cytochrome C (Cyt-C) cytosolic/mitochondrial (B), Bcl-2/Bax (C), cleaved caspase-3 (D) and cleaved caspase-9 (E) in H9C2 cells in each group; *, *P*<0.05 compared to Control group; #, *P*<0.05 compared to the hypoxia/ reoxygenation (H/R) group; and, *P*<0.05 compared to the H/R+anti-miR-155 group

***The activities of CK and LDH in the serum of rats and H9C2 cells***

As compared to Sham group, rats in the I/R group had the elevated serum levels of CK, LDH, cTnT and cTnI (all *P*< 0.05, Figure 2A, B). Meanwhile, I/R+anti-miR-155 group was significantly lower than the I/R group regarding the levels of CK, LDH, cTnT and cTnI (all *P*< 0.05), while I/R+anti-control group had no significant difference from the I/R group in the four indexes (all *P* > 0.05). Besides, the serum levels of CK, LDH, cTnT and cTnI were higher in rats from I/R+anti-miR-155+shHIF-1α group by comparison with those from the I/R+anti-miR-155 group (all *P*<0.05). On the other hand, the activities of CK and LDH in H9C2 cells of different treatment groups were also detected (Figure 2C), which were significantly enhanced in H9C2 cells induced by H/R (all *P*<0.05). Moreover, H9C2 cells in H/R+anti-miR-155 group demonstrated an obvious reduction in CK and LDH activities compared to those in H/R group, while these two indexes in the cells in H/R+anti-miR-155+shHIF-1α group was apparently higher than those in the H/R+anti-miR-155 group (all *P*<0.05). 

***Apoptosis detection in myocardial tissues and H9C2 cells***

When compared to the Sham rats and Control cells respectively, both the myocardial tissues of I/R-induced rats and H/R-induced H9C2 cells showed significant increase in apoptosis rate (all *P*<0.05, Figure 3). However, the apoptosis rate was reduced in the myocardial tissues of I/R-induced rats and H/R-induced H9C2 cells after treatment with anti-miR-15 (all *P*<0.05). Furthermore, the myocardial tissues of rats from the I/R+anti-miR-155+shHIF-1α group had higher apoptosis rate than those from the I/R+anti-miR-155 group (all *P*<0.05). Similarly, apoptosis rate in H9C2 cells from the H/R+anti-miR-155+shHIF-1α group were higher than those from the H/R+anti-miR-155 group (all *P*<0.05). 

***Expression of miR-155 and HIF-1α in rats and H9C2 cells***

As shown in Figure 4, the expression level of miR-155 detected by qRT-PCR was increased in the myocardial tissues of I/R-induced rats and H/R-induced H9C2 cells, but was decreased with the treatment of anti-miR-155 (all *P*<0.05). Besides, the expression of HIF-1α was also determined *in vitro* and *in vivo* via Western blotting. Obviously, the protein expression of HIF-1α was decreased in the myocardial tissues of I/R-induced rats and H/R-induced H9C2 cells, but was increased with anti-miR-155 treatment (all *P*<0.05). Moreover, shHIF-1α treatment could reverse the promoting effect of anti-miR-155 on HIF-1α expression. 

***HIF-1α*** **is the target gene of miR-155**

 The targetscan website was used to analyze the targeting relationship between miR-155 and HIF-1α, as illustrated in Figure 5, and the result demonstrated that miR-155 could bind to the 3’-UTR of *HIF-1α* mRNA. According to the dual-luciferase reporter gene assay, the luciferase activity of *HIF-1α*-3’UTR-WT was significantly reduced when transfected with miR-155 mimic (*P*< 0.05), but showed no significant difference from the *HIF-1α*-3’UTR-MUT group after transfection with miR-155 mimic (*P*>0.05), as compared to the NC group. These results indicated that *HIF-1α* was the direct target gene of miR-155.

***Determination of MMP and cell viability in H9C2 cells***

As shown in Figure 6, the decreased ratio of red/green fluorescence was observed in H9C2 cells in the H/R group when compared to Control group (*P* < 0.05), suggesting that the mitochondrial membrane potential (MMP) in H9C2 cells was declined after H/R induction. Besides, the ratio of red/green fluorescence in cells in the H/R+anti-miR-155 group was higher than those in the H/R group, but the ratio of red/green fluorescence and MMP were significantly lower in cells from the H/R+anti-miR-155+shHIF-1α group than those from H/R+anti-miR-155 group (all *P*<0.05). Additionally, we used MTT assay to evaluate the cell viability in each group and found that the H/R-induced H9C2 cells presented the reduced viability than Controls (*P*< 0.05), while those H/R-induced H9C2 cells treated with anti-miR-155 exhibited higher cell viability (*P*< 0.05). Furthermore, H9C2 cells in the H/R+anti-miR-155+shHIF-1α group demonstrated the reduced H9C2 cell viability (*P*<0.05), whereas no significant difference was observed in the H/R+anti-control group in terms of MMP and cell viability, as compared to H/R group (all *P*>0.05). 

***Expression of apoptosis-related proteins in H9C2 cells***

After H/R-induced injury, a lot of Cyt-C was released into the cytoplasm from mitochondria, and the ratio of Cyt-C cytosolic/mitochondria increased apparently with up-regulation of cleaved caspase-3 and cleaved caspase-9 and declined ratio of Bcl-2/Bax (all *P*<0.05, Figure 7). Conversely, the H/R-induced H9C2 cells treated with anti-miR-155 showed significant decreases in ratio of Cyt-C cytosolic/mitochondria, and expression levels of cleaved caspase-3 and cleaved caspase-9, and an obvious increase in the ratio of Bcl-2/Bax (all *P*<0.05). However, as compared to the H/R+anti-miR-155 group, H9C2 cells in the H/R+anti-miR-155+shHIF-1α group had the increased Cyt-C cytosolic/mitochondria ratio and elevated expression levels of cleaved caspase-3 and cleaved caspase-9, as well as decreased Bcl-2/Bax ratio (all *P*<0.05). Moreover, there was no difference between H/R+anti-control group and H/R group concerning the above indexes (all *P*>0.05). 

## Discussion

In the current research, we have revealed an important finding that inhibiting miR-155 could significantly reduce the IA of experimental I/R rats through establishing the rat models with myocardial I/R injury. Similar to a previous study, suppression of endogenous miR-155 could attenuate cardiac hypertrophy and remodeling and prevent the progression of heart failure, ultimately improving the survival rate of rats (23). Knock-out of miR-155 could protect the cardiac fibroblasts in a previous study, which could inhibit cardiac fibroblasts growth and promote cardiac remodeling via targeting TP53INP1, becoming a new therapeutic method for cardiac remodeling (24). When myocardial I/R injury occurred, the cell membrane permeability as well as the enzymes involved in myocytes injury like CK and LDH, which would leak from myocardial tissues to the blood would be increased (25, 26). Meanwhile, the H/R-induced H9C2 cell models were also constructed to further confirm our result. As such, the activities of CK and LDH were determined* in vivo* and *in vitro* in the present study, since they could act as crucial factors to identify the cell injury and membrane integrity. Consequently, the CK and LDH activities in the serum of I/R rats and H/R-induced H9C2 cells were remarkably increased, which, however, were rescued by anti-miR-15 treatment, suggesting that inhibition of miR-155 may protect myocardial injury from I/R in rats and H/R in H9C2 cells.

In addition, apoptosis was thought to have an essential effect on the spectrum of myocardial damage (27), so we detected the myocardial cell apoptosis via rat models *in vivo* and cell models *in vitro*. As a result, we found that the cell apoptosis rate was significantly elevated in both the myocardial tissues of I/R-induced rats and H/R-induced H9C2 cells, whereas anti-miR-155 treatment led to the reduced cell apoptosis. Consistent with our findings, Xing *et al. *also reported that miR-155 inhibitors could reduce the cell apoptosis via targeting Ras homolog enriched in brain (Rheb) to decrease the infarction size of rats with ischemic stroke (28), which partially suggested that the protective role of anti-miR-155 in I/R injury would be performed through reducing myocardial cell apoptosis. Besides, the opening of mitochondrial permeability transition pore (mPTP) was widely accepted as a critical mediator of myocardial reperfusion injury, which could destroy the integrity of the mitochondrial membrane to induce the release of pro-apoptotic factors and Cyt C and promote caspase-3 activation to induce cell apoptosis, consequently resulting in the reduction of MMP (29-31). After determination of the MMP of H9C2 cells in our experiment, we found the MMP in H9C2 cells was declined after H/R induction, but was enhanced with the treatment of anti-miR-155. Furthermore, anti-miR-155 treatment could also increase the protein expressions of cleaved caspase-3, cleaved caspase-9, and the ratio of Bcl-2/Bax, with the decreased Cyt-C cytosolic/mitochondria ratio in H/R-induced H9C2 cells, according to Western blotting. As reported, the release of Cyt-C across the outer mitochondrial membrane into cytoplasm led to the apparent increased ratio of Cyt-C cytosolic/mitochondria and activated Cyt c-caspase-3 cascade activation, eventually causing the cell apoptosis (32, 33). Bcl-2 protein, mainly distributed in mitochondrial inner membrane, can modulate the opening of mPTP to block the release of Cyt-C, thus playing a significant inhibitory role in cell apoptosis (34). Meanwhile, Bax can influence the mPTP to promote the release of Cyt-C (35, 36). As suggested by Wang *et al.* suppression of miR-155 can attenuate lipopolysaccharides (LPS)-induced myocardial cell apoptosis, increase Bcl-2, reduce Bax, and improve heart function, which constituted a new method for controlling of septic myocardial dysfunction (37), showing that down-regulating miR-155 may underlie the cardioprotection elicited by inhibition of myocardial cell apoptosis mediated by mitochondrial pathway.

On the other hand, HIF-1α was turned out to be the target gene of miR-155, as predicted and identified by dual-luciferase reporter gene assay. In our models, HIF-1α shRNA could reverse the protective role of anti-miR-155 in I/R-induced rats and H/R-induced H9C2 cells. In agreement with this, Bruning *et al. *also confirmed the targeting relationship between miRNA-155 and HIF-1α in ischemic cells (38). HIF-1, widely distributed in mammalian tissues and cells, is a transcriptional regulator for hypoxic response (39), which is a heterodimer consisted of α (an oxygen-regulated) and β (a constitutively expressed) subunits, and its bioactivity mainly depends on α subunit (40). Under nonmaximal conditions, HIF-1α in cytoplasm was modified by proline hydroxylase (PHD) and degraded and deactivated via the ubiquitination pathway; however, under hypoxia condition, HIF-1α would enter the nucleus to form a heterodimer with β subunit, which can bind to hypoxia response element to regulate the transcription and activation of over 200 downstream target genes, and thereby promoting the cell survival (41). Moreover, the highly-expressed HIF-1α in myocardial I/R injury could reduce the oxidative stress, as revealed by Ong *et al.* and inhibit the opening of mPTP, thus achieving protection against I/R-induced injury (42). In the study of Zhou *et al.* sevoflurane could activate HIF-1α expression and inhibit caspase-3 expression to ameliorate myocardial I/R injury in the heart of rats (43). Not surprisingly, our study also showed that treatment with anti-miR-155 in both the I/R-induced rats and H/R-induced H9C2 cells had significant decrease in miR-155 expression and obvious increase in HIF-1α expression, which supported the hypothesis that the protective role of anti-miR-155 in I/R injury was closely associated with its function in elevating HIF-1α expression. 

## Conclusion

Our study indicated that the inhibition of miR-155 can maintain the MMP and inhibit myocardial cell apoptosis mediated by mitochondria-pathway through specifically targeting HIF-1α, thereby alleviating the myocardial injury, which contributed to provide a new therapeutic clue for the clinical treatment of I/R injury. 
